# Association between carbon monoxide poisoning and adrenal insufficiency: a nationwide cohort study

**DOI:** 10.1038/s41598-022-20584-y

**Published:** 2022-09-28

**Authors:** Chien-Cheng Huang, Chung-Han Ho, Yi-Chen Chen, Chien-Chin Hsu, Hung-Jung Lin, Jhi-Joung Wang, Shih-Bin Su, How-Ran Guo

**Affiliations:** 1 Department of Emergency Medicine, Chi Mei Medical Center, 901 Zhonghua Road, Yongkang District, Tainan City, 710 Taiwan; 2 Department of Emergency Medicine, Kaohsiung Medical University, Kaohsiung, Taiwan; 3 Department of Medical Research, Chi Mei Medical Center, Tainan, Taiwan; 4 Department of Information Management, Southern Taiwan University of Science and Technology, Tainan, Taiwan; 5 Department of Emergency Medicine, Taipei Medical University, Taipei, Taiwan; 6 Department of Anesthesiology, Chi Mei Medical Center, Tainan, Taiwan; 7 Department of Anesthesiology, National Defense Medical Center, Taipei, Taiwan; 8 Department of Leisure, Recreation and Tourism Management, Southern Taiwan University of Science and Technology, Tainan, Taiwan; 9 Department of Medical Research, Chi Mei Medical Center, Liouying, Tainan, Taiwan; 10 Department of Occupational Medicine, Chi Mei Medical Center, Tainan, Taiwan; 11 Department of Occupational and Environmental Medicine, National Cheng Kung University Hospital, Tainan, Taiwan; 12 Occupational Safety, Health, and Medicine Research Center, National Cheng Kung University, Tainan, Taiwan; 13 Department of Environmental and Occupational Health, College of Medicine, National Cheng Kung University, 138 Sheng-Li Road, Tainan, 704 Taiwan

**Keywords:** Endocrine system and metabolic diseases, Endocrine system and metabolic diseases, Environmental sciences, Health care, Health occupations

## Abstract

Carbon monoxide poisoning may damage the brain and adrenal glands, but it is unclear whether it is associated with adrenal insufficiency. We identified all COP patients diagnosed between 1999 and 2012 in Taiwan using the Nationwide Poisoning Database and selected a reference cohort (participants without COP) from the same database by exact matching of age and index date at a 1:2 ratio. Participants with a history of adrenal insufficiency or steroid use of more than 14 days were excluded. We followed up participants until 2013 and compared the risk of developing adrenal insufficiency between the two cohorts. The 21,842 COP patients had a higher risk for adrenal insufficiency than the 43,684 reference participants (adjusted hazard ratio [AHR] = 2.5; 95% confidence interval [CI]: 1.8–3.5) after adjustment for sex and underlying comorbidities (liver disease, thyroid disease, mental disorder). The risk continued to elevate even after 1 year (AHR = 2.1; 95% CI: 1.4–3.4). The COP patients who had acute respiratory failure had an even higher risk for adrenal insufficiency than those without acute respiratory failure, which may indicate a dose–response relationship. Stratified analyses showed that female patients had an elevated risk (AHR = 3.5; 95% CI: 2.1–6.0), but not male patients. Younger patients (< 50 years) had higher risks, and the AHR reached statistical significance in the age groups 20–34 (AHR = 5.5; 95% CI: 1.5–20.6) and 35–49 (AHR = 4.9; 95% CI: 2.3–10.6) years old. The risk for developing adrenal insufficiency elevated after COP, especially in female and younger patients. Carbon monoxide is the most common gaseous agent causing acute intoxication worldwide. Results of the current study call for monitoring adrenal function of patients with COP.

## Introduction

Carbon monoxide (CO) is a product of incomplete combustion of organic matter and has a nearly 250-fold affinity to hemoglobin compared with oxygen, which may cause hypoxia of tissues^1^. Carbon monoxide poisoning (COP) is an important public health issue because it contributes to many accidental and suicidal deaths worldwide^2^. The brain and heart are the most organs susceptible to hypoxia after COP due to their high metabolite rates^3–6^, and therefore, neurologic sequelae and mortality are the major complications following COP^7–13^.

In addition to hypoxia, COP may also induce immunological and inflammatory reactions in organs, including those that may lead to endocrine disorders^9,14^ and autoimmune connective tissue disease^15^, by producing reactive oxygen species^1,9,16^. A recent nationwide study in Taiwan found that the risk of developing diabetes increased by nearly 2 folds after COP^9^. The combination of damages to the brainstem, hypothalamus, and pancreas was suspected to be the cause^9^. Adrenal glands, which are located at the top of kidneys, produce three classes of hormones: glucocorticoids, mineralocorticoids, and androgens^17,18^. The secretion of these hormones is regulated by the brain, more specifically, the hypothalamus and pituitary gland^18,19^. The “hypothalamic–pituitary–adrenal axis” refers to the pathway through which hypothalamus and pituitary gland regulate the production of cortisol—the main glucocorticoid secreted by adrenal glands^18,20^. Any dysfunction in the pathway, including those in the brain and adrenal gland that can possibly be caused by COP, may contribute to adrenal insufficiency. However, when we conducted a search using “carbon monoxide poisoning” and “adrenal insufficiency” as keywords in PubMed and Google Scholar, we did not find any studies on this subject. Therefore, we carried out a study to fill the data gap.

## Materials and methods

### Data sources

We used two data subsets of the Taiwan National Health Insurance Research Database (NHIRD), which covers nearly 100% of the population in Taiwan^21^. The Nationwide Poison Database (NPD) contains claim data on all the poisonings including COP between 1999 and 2013 in Taiwan. The Longitudinal Health Insurance Database 2000 (LHID2000) contains all claim data on 1,000,000 individuals randomly selected from the original NHIRD^22^. The NHIRD is maintained by the National Health Research Institutes and provided to scientists in Taiwan for research purposes^21^.

### Study design, setting, and participants

**Figure 1:**
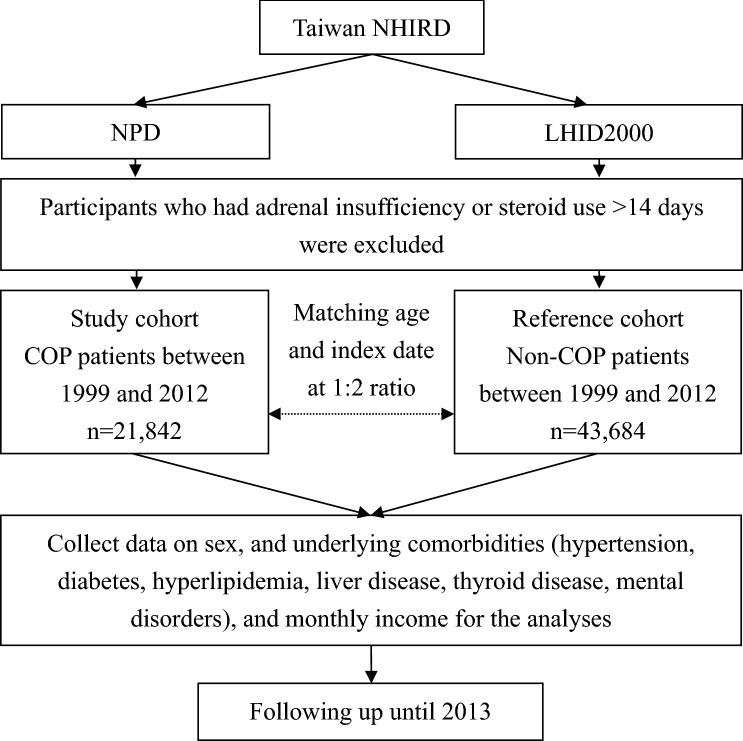
Flowchart of this study. NHIRD, National Health Insurance Research Database; NPD, Nationwide Poison Database; LHID2000, Longitudinal Health Insurance Database 2000.

We conducted a nationwide population-based cohort study and identified all the participants diagnosed with COP between 1999 and 2012 as the study cohort from the NPD (Fig. 1). The reference cohort was made up of participants without COP who were selected from LHID2000 by exact matching of ages and index dates with those of COP participants at a 1:2 ratio. The index date was defined as the date of admission or visit to the emergency department of the COP patients. The treatment of COP in Taiwan is 100% oxygen immediately and identify risks for possible referral for hyperbaric oxygen treatment according to the guidelines set by American College of Emergency Physicians^23^.

### Definitions of the variables

The COP patients were defined as the participants with diagnoses identified by International Classification of Diseases, Ninth Revision, Clinical Modification (ICD-9-CM) codes of 986 (toxic effect of carbon monoxide), E868 (accidental poisoning by unspecified carbon monoxide), E952 (suicide and self-inflicted poisoning by other specified gases and vapors), or E982 (poisoning by other gases undetermined whether accidentally or purposely inflicted) during either at hospital admission or during a visit to the emergency department. All the participants who had adrenal insufficiency (ICD-9-CM codes: 255.4 or 255.5) or steroid use (Anatomical Therapeutic Chemical [ATC] codes: A07EA01, C05AA04, D07AA03, D07XA02, H02AB06, H02AB06, R01AD02, S01BA04, S01CB02, S02BA03, or S03BA02) of more than 14 days before the index date were excluded. A case of adrenal insufficiency was defined as having the ICD-9-CM code of 255.4 or 255.5 as one of the main diagnoses in at least one hospitalization or three ambulatory care claims. The National Health Insurance conducts routine scrutiny of the claims and imposes a 500-fold fine on claims for improper treatment or diagnosis and presses criminal charge on falsified claims. Therefore, the diagnoses should be valid under our definition, which has been used in other studies^24^. The diagnosis of adrenal insufficiency was made by the treating physicians, generally based on the following: (1) measurement of the blood cortisol level; (2) measurement of adrenocorticotropic hormone (ACTH) for deciding the level of defect (i.e., primary, secondary, or tertiary); (3) determination of the causes of the defect; and (4) evaluation of the associated problems^18^. The age subgroups were defined as < 20, 20–34, 35–49, 50–64, and ≥ 65 years. We adjusted for the effects of underlying comorbidities including liver disease (ICD-9-CM 570–576), thyroid disease (ICD-9-CM 240, 241, 242, 244, 245, 246, and 193), and mental disorders (ICD-9-CM 290–302 and 306–319) because they are risk factors for adrenal insufficiency and thus potential confounders of this study^20^. The monthly income brackets were defined as < 20,000, 20,000–40,000, and > 40,000 New Taiwan dollars (NTD).

### Comparison of the risk of adrenal insufficiency between the two cohorts

We compared the risk of adrenal insufficiency between the two cohorts by following up all participants until 2013. Separate follow-ups within 1 year and more than 1 year after COP were also performed to evaluate the short- and long-term term effects. We also performed stratified analyses according to age and sex to evaluate whether they were effect modifiers.

### Ethical statement

This study was approved by the Institutional Review Board at Chi Mei Medical Center and strictly conducted according to the Declaration of Helsinki. Because the NHIRD contains de-identified information of the participants, informed consent from the participants was waived, which did not affect the rights and welfare of the participants.

### Statistical methods

The standardized difference was used to illustrate the acceptable balance between the COP and matched cohorts in demographic characteristics, underlying comorbidities, and monthly income. An absolute standardized difference of > 0.1 was considered to indicate imbalance^25^. Because COP is often fatal in Taiwan, we used competing risk survival analyses to compare the risk of adrenal insufficiency between the two cohorts. Cumulative incidences for all-cause mortality and adrenal insufficiency in both cohorts were calculated. Following bivariable analyses, we performed multivariable analyses with adjustment for sex and underlying comorbidities (including liver disease, thyroid disease, and mental disorders). Because we matched age in selecting the reference cohort, we did not include age in the multivariable analyses. The risk of adrenal insufficiency was estimated using Cox proportional hazard regression analysis with aggregated sandwich estimator to calculate the adjusted hazard ratio of adrenal insufficiency between the COP and matched cohorts. To investigate the association between severity of COP and occurrence of adrenal insufficiency (dose–response relationship), we used acute respiratory failure (ARF) as an indicator of severity^23^. We defined a case of ARF as having the ICD-9-CM diagnostic code 518.81 or 518.84, receiving endotracheal intubation, using of bi-level positive airway pressure. All patients were right-censored to the new-onset adrenal insufficiency, the date of death, or the end of follow-up date, December 31, 2013. We used the Kaplan–Meier’s method and the log-rank test to compare the risk for adrenal insufficiency between the two cohorts during the follow-up period. All the analyses were performed using SAS 9.4 for Windows (SAS Institute, Cary, NC, USA) at a two-tailed significance level of 0.05.

## Results

In total, we identified 21,842 COP patients during the study period and selected 43,684 references accordingly in this study. The mean age was 36.2 (standard deviation = 15.4) years, and 82.5% of COP patients were younger than 50 years (Table 1). The sex ratio within COP patients was close to unity. Lower monthly income was more common in COP patients. The mean (standard deviation) of follow-up period in COP and reference cohorts were 5.0 (3.5) years and 5.4 (3.4) years, respectively.

**Table 1:** Demographic characteristics, underlying comorbidities, and monthly income in COP and reference cohorts.

Variable	COP (n = 21,842)	Reference (n = 43,684)	Standardized difference**
Age (year)	36.2 ± 15.4	36.2 ± 15.4	< 0.1
Age (year)			
< 20	2470 (11.3)	4942 (11.3)	< 0.1
20–34	8628 (39.5)	17,256 (39.5)	
35–49	6932 (31.7)	13,863 (31.7)	
50–64	2678 (12.3)	5355 (12.3)	
≥ 65	1134 (5.2)	2268 (5.2)	
Sex			
Female	10,970 (50.2)	22,733 (52.0)	< 0.1
Male	10,872 (49.8)	20,951 (48.0)	
Underlying comorbidity			
Hypertension	2340 (10.7)	4310 (9.9)	< 0.1
Diabetes	1242 (5.7)	2069 (4.7)	< 0.1
Hyperlipidemia	1613 (7.4)	3005 (6.9)	< 0.1
Liver disease	2856 (13.1)	4941 (11.3)	< 0.1
Thyroid disease	740 (3.4)	2403 (5.5)	0.1
Mental disorder	6350 (29.1)	9902 (22.7)	0.1
Monthly income (NTD)			
< 20,000	15,780 (72.3)	27,341 (62.6)	0.2
20,000–40,000	4840 (22.2)	12,127 (27.8)	
> 40,000	1222 (5.6)	4216 (9.7)	
Follow-up period (year)			
Mean (standard deviation)	5.0 (3.5)	5.4 (3.4)	0.1
Median (Q1–Q3)	4.7 (2.0–7.7)	5.2 (2.6–7.9)	

*COP* carbon monoxide poisoning; *NTD* New Taiwan Dollars. Data are expressed as mean ± standard deviation or n (%). **Take absolute value.

**Figure 2:**
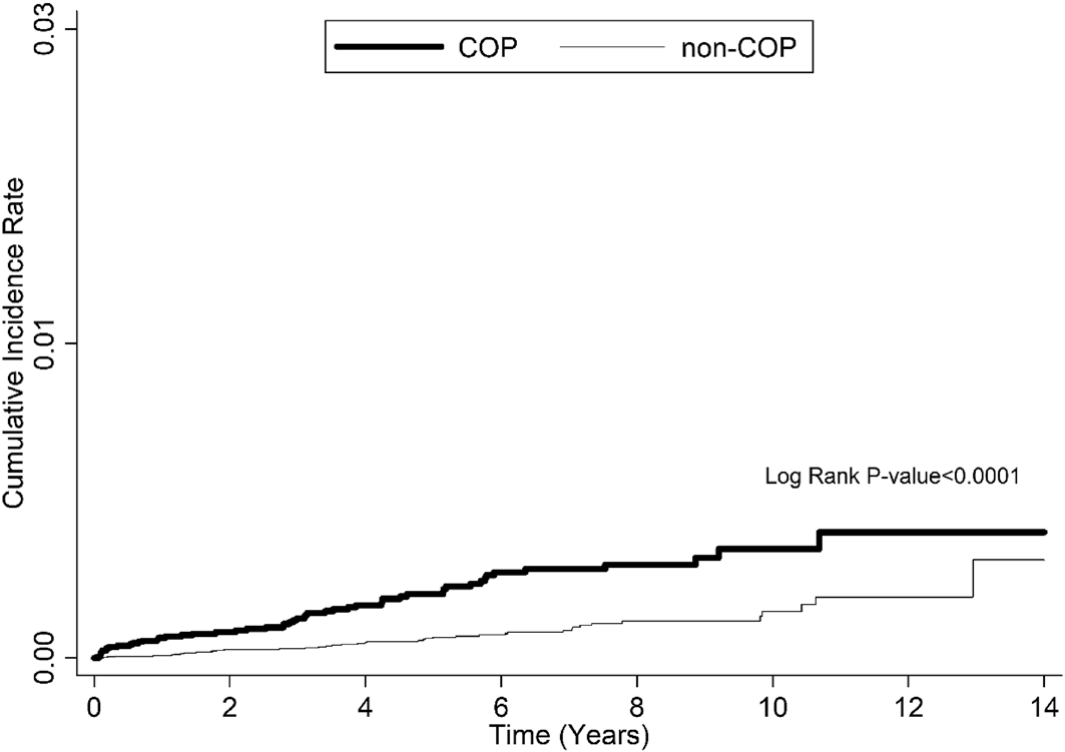
Comparison of the risk of adrenal insufficiency between the COP and reference (non-COP) cohorts during follow-up by Kaplan–Meier’s method and the log-rank test.

During the follow-up, COP patients had a risk for adrenal insufficiency than the references with a hazard ratio (HR) of 2.6 (95% confidence interval [CI]: 1.7–3.9) after taking competing risk into account (Table 2). Kaplan–Meier’s method and the log-rank test showed a higher risk for adrenal insufficiency in the COP patients than in the references (Fig. 2). After adjusting for sex, liver disease, thyroid disease, and mental disorder, we found the adjusted hazard ratio (AHR = 2.5) was like the crude HR (2.6), with a 95% CI like that in the initial analysis (1.8–3.5). In stratified analyses, we found patients under 50 years of age had higher risks, and the AHR reached statistical significance in the age groups 20 − 34 (AHR = 5.5; 95% CI: 1.5–20.6) and 35 − 49 (AHR = 4.9; 95% CI: 2.3–10.6) years old. The increased risk was more prominent in the women than in the men (AHR: 3.5 vs. 1.6). In the reference cohort, however, the cumulative incidence rate was 0.1% in both sexes. The increases in the risk were more prominent within 1 year of follow-up (AHR = 8.2; 95% CI: 2.6–25.4) but remained statistically significant afterwards (AHR = 2.1; 95% CI: 1.4–3.4). When we conducted the analyses on COP patients with ARF (i.e., a subgroup of more severe COP), we found that this population had an AHR of 9.9 (95% CI: 1.2–85.2) even after more than 1 year of follow-up. The mean (standard deviation) of inpatient visits within 1 year of follow-up in COP and reference cohorts were 1.8 (1.7) and 1.4 (1.2), respectively (Supplemental Table 1). The mean (standard deviation) of outpatient visits within 1 year of follow-up in COP and reference cohorts were 17.0 (16.8) and 13.2 (13.1), respectively.

**Table 2:** Comparison of the risk for adrenal insufficiency during whole the follow-up period between the COP and references cohorts using competing risk survival analyses.

Variable	COP cohort N (%)		Reference cohort N (%)		Crude HR (95% CI)	AHR (95% CI)†
	All-cause mortality	Adrenal insufficiency	All-cause mortality	Adrenal insufficiency		
Overall analysis	2826 (12.9)	53 (0.2)	1203 (2.8)	41 (0.1)	2.6 (1.8–3.5)*^#^	2.5 (1.8–3.5)*^#^
Stratified analyses						
Age (year)						
< 20	86 (3.5)	3 (0.1)	26 (0.5)	1 (< 0.01)	6.0 (0.6–57.6)	5.0 (0.5–47.4)
20–34	818 (9.5)	9 (0.1)	201 (1.2)	3 (< 0.01)	6.0 (1.6–22.0)*	5.5 (1.5–20.6)*
35–49	951 (13.7)	22 (0.3)	249 (1.8)	9 (0.01)	4.9 (2.2–10.6)*	4.9 (2.3–10.6)*
50–64	522 (19.5)	10 (0.4)	206 (3.9)	12 (0.2)	1.7 (0.7–3.9)	1.9 (0.8–4.3)
≥ 65	449 (39.6)	9 (0.8)	521 (23.0)	16 (0.7)	1.1 (0.5–2.6)	1.1 (0.5–2.6)
Sex						
Female	1053 (9.6)	36 (0.3)	461 (2.0)	21 (0.1)	3.5 (2.1–6.1)*	3.5 (2.1–6.0)*
Male	1773 (16.3)	17 (0.2)	742 (3.5)	20 (0.1)	1.6 (0.9–3.1)	1.6 (0.8–3.0)
Follow-up period						
≤ 1 year	1528 (7.1)	16 (0.1)	253 (0.6)	4 (0.01)	9.3 (2.7–32.5)*	8.2 (2.6–25.4)*
> 1 year	1298 (5.9)	37 (0.2)	950 (2.2)	37 (0.1)	2.1 (1.2–3.4)*	2.1 (1.4–3.4)*

*COP* carbon monoxide poisoning; *HR* hazard ratio; *AHR* adjusted hazard ratio; *CI* confidence interval. *p < 0.05. †Adjusted for sex, underlying comorbidities including liver disease, thyroid disease, and mental disorder. ^#^Cox regression model with robust sandwich approach.

In follow-up within 1-year, stratified analyses showed that female COP patients had a higher risk for adrenal insufficiency than female references, with an AHR of 23.8 (95% CI: 3.2–177.1) (Table 3). However, the increased risk in male COP patients did not reach statistical significance (AHR = 3.2; 95% CI: 0.7–15.4). The number of participants developed adrenal insufficiency within the first year of follow-up was too small for conducting stratified analyses by age. After more than 1 year of follow-up, female COP patients still had a higher risk for adrenal insufficiency than their references (AHR = 2.8; 95% CI: 1.5–4.9), but male COP patients did not (AHR = 1.4; 95% CI: 0.7–3.0) (Table 4). In the age subgroups, those under 50 years of age had higher risks, and the AHR associated with the age groups 20–34 years (4.2; 95% CI: 1.2–15.2) and 35–49 years (3.7; 95% CI: 1.7–8.2) reached statistical significance.

**Table 3:** Comparison of the risk for adrenal insufficiency at follow-up within 1 year between the COP and reference cohorts using competing risk survival analyses.

Variable	COP cohort N (%)		Reference cohort N (%)		Crude HR (95% CI)	AHR (95% CI)†
	All-cause mortality	Adrenal insufficiency	All-cause mortality	Adrenal insufficiency		
Overall analysis	1528 (7.1)	16 (0.1)	253 (0.6)	4 (0.01)	9.3 (2.7–32.5)*	8.2 (2.6–25.4)*
Stratified analyses						
Age (year)						
< 20	53 (2.2)	1 (0.04)	3 (0.1)	0 (0.0)	–	–
20–34	440 (5.1)	2 (0.02)	49 (0.3)	0 (0.0)	–	–
35–49	537 (7.8)	6 (0.1)	55 (0.4)	0 (0.0)	–	–
50–64	309 (11.5)	3 (0.1)	43 (0.8)	2 (0.04)	3.0 (0.5–18.0)	3.0 (0.5–18.8)
≥ 65	189 (16.7)	4 (0.4)	103 (4.5)	2 (0.1)	8.0 (0.9–71.6)	4.4 (0.7–30.3)
Sex						
Female	569 (5.2)	11 (0.1)	92 (0.4)	1 (< 0.01)	18.7 (2.4–147.2)*	23.8 (3.2–177.1)*
Male	959 (8.8)	5 (0.1)	161 (0.8)	3 (0.01)	4.8 (0.9–24.8)	3.2 (0.7–15.4)

COP, carbon monoxide poisoning; HR, hazard ratio; AHR, adjusted hazard ratio; CI, confidence interval. *p < 0.05. †Adjusted for sex, underlying comorbidities including liver disease, thyroid disease, and mental disorder.

**Table 4:** Comparison of the risk for adrenal insufficiency after more than 1 year of follow-up between the COP and reference cohorts using competing risk survival analyses.

Variable	COP cohort N (%)		Reference cohort N (%)		Crude HR (95% CI)	AHR† (95% CI)
	All-cause mortality	Adrenal insufficiency	All-cause mortality	Adrenal insufficiency		
Overall analysis	1298 (5.9)	37 (0.2)	950 (2.2)	37 (0.1)	2.1 (1.2–3.4)*	2.1 (1.4–3.4)*
Stratified analyses						
Age (year)						
< 20	33 (1.3)	2 (0.1)	23 (0.5)	1 (0.02)	4.1 (0.4–45.0)	3.4 (0.3–36.6)
20–34	378 (4.4)	7 (0.1)	152 (0.9)	3 (0.02)	4.9 (1.3–18.8)*	4.2 (1.2–15.2)*
35–49	414 (6.0)	16 (0.2)	194 (1.4)	9 (0.1)	3.5 (1.5–8.5)*	3.7 (1.7–8.2)*
50–64	213 (8.0)	7 (0.3)	163 (3.0)	10 (0.2)	1.3 (0.4–3.7)	1.8 (0.7–4.8)
≥ 65	260 (22.9)	5 (0.4)	418 (18.4)	14 (0.6)	0.5 (0.1–2.4)	0.8 (0.3–2.1)
Sex						
Female	484 (4.4)	25 (0.2)	369 (1.6)	20 (0.1)	3.2 (1.6–6.1)*	2.8 (1.5–4.9)*
Male	814 (7.5)	12 (0.1)	581 (2.8)	17 (0.1)	1.0 (0.4–2.4)	1.4 (0.7–3.0)

*COP* carbon monoxide poisoning; *HR* hazard ratio; *AHR* adjusted hazard ratio; *CI* confidence interval. *p < 0.05. †Adjusted for sex, underlying comorbidities including liver disease, thyroid disease, and mental disorder.

In the comparison between patients with and without ARF in the COP cohort, we found that COP patients with ARF had a higher risk of adrenal insufficiency than COP patients without ARF (AHR: 2.1; 95% CI: 1.0–4.5) (Supplementary Table 2). The difference in the risk for adrenal insufficiency between male and female COP patients with ARF did not reach statistical significance (Supplementary Table 3). To investigate the association between severity of COP and adrenal insufficiency, we also performed subgroup analysis according to ICU admission, hospitalization, and outpatient visit (Supplementary Table 4). The results showed that there was no significant difference for developing adrenal insufficiency among the three subgroups.

## Discussion

This study showed that the COP cohort had a higher risk for adrenal insufficiency than the reference cohort during the entire follow-up period, but the increases in the risk were more prominent during the first year and in women and younger population. The COP patients who had ARF had an even higher risk for adrenal insufficiency than COP patients without ARF, and this dose–response relationship supported a causal relationship. This association might be attributable to the hypoxic injury, inflammation, and immunological reaction in the brain and adrenal glands following COP.

Hypoxic injury in the brain and adrenal glands may be the major cause of adrenal insufficiency observed in this study. Because this finding is novel, there is no direct evidence in the literature to support it. Previous studies reported that hypoxia is involved in endocrine organ development, hormone regulation, and endocrine disorders^26^. The hypoxic effect is due to hypoxia-inducible factor (HIF)-dependent and HIF-independent pathways^26^. In addition to hypoxia, inflammation and immunological reaction are suspected to be the cause of neurologic deficits following COP^27^. The inflammatory mechanism of COP includes activation of platelet by displacing platelet nitric oxide, peroxynitrite production, inhibition of mitochondrial function, production of reactive oxygen species, release of free heme, ensuing increase of heme oxygenase (HO)-1, and oxidative stress^27^. More study is needed to clarify the mechanism between COP and adrenal insufficiency.

We found that females were more susceptible to adrenal insufficiency following COP than males. In the literature, there is no sex-based difference in the occurrence of adrenal insufficiency^18^, and our observation of similar cumulative incidence between the two sexes in the reference cohort (both were 0.1%) is compatible with the literature. A recent study on the long-term mortality after COP reported a similar phenomenon; i.e. the increased risk was more prominent in women (incidence rate ratio [IRR] = 8.43; 95% CI: 4.64–15.32) than in men (IRR = 4.07; 95% CI: 2.52–6.57)^7^. However, the reason why females are more susceptible to COP is unclear.

We also found that younger populations were more susceptible to adrenal insufficiency following COP. A recent study on the long-term mortality after COP reported a similar phenomenon; i.e. the increases in the risk were more prominent in younger patients than in older patients (IRR = 15.62 in 0–20 years vs. IRR = 10.64 in 30–49 years vs. IRR = 3.49 in ≥ 50 years)^7^. A possible explanation is that younger people have less risk factor for adrenal insufficiency than older people, and therefore the role of COP in adrenal insufficiency is relatively more important in the younger population.

This study revealed that the increased risk of adrenal insufficiency was more prominent within the first year of follow-up than that afterwards. The finding suggests that the impact of COP on adrenal insufficiency is larger in the short term and decreases with time. There is no direct evidence in the literature about this, but a study reported that adrenal insufficiency is a complication of immune checkpoint inhibitor treatment for cancer, and that patients recovered from it after stopping the medication and receiving steroid supply^28^. The possible mechanism is the accommodation of hypothalamic‐pituitary‐adrenal axis after cessation of COP. More evidence is needed to validate this speculation. Nonetheless, it is also possible that COP patients may have been diagnosed with adrenal insufficiency earlier than the reference cohort by continuing to visit the hospital for other complications during the early days of follow-up period, because the inpatient and outpatient visits in the COP cohort within 1 year of follow-up were more than those in the reference cohort.

Despite the novel findings of this study and its nationwide population-based design, it has some limitations. First, some risk factors for adrenal insufficiency, such as hereditary factors, were not accounted for in this study. We did include patients with congenital adrenal hyperplasia as well as pituitary tumors, adrenal hemorrhage, and tuberculosis in the preliminary analyses (data not shown) but found that the numbers of such cases were very small in our study population. Therefore, these factors are unlikely to introduce remarkable biases to our results. Second, although this was a nationwide study, the case number of adrenal insufficiencies was still small. Consequently, some of the AHR did not reach statistical significance even though the value is relatively large, and we were unable to conduct stratified analyses by age within the first year of follow-up. Third, carboxyhemoglobin levels and exposure duration of COP were not available in this study. However, many studies reported that the carboxyhemoglobin level is neither a good indicator of disease severity nor a predictor of prognosis^23,29,30^. Fourth, it is difficult to determine causality due to the limitations of the study design. Fifth, in addition to steroids, adrenal insufficiency may be induced by other medications or substances, particularly in the COP patients with suicide attempt. The database we used did not include information on the medication or substances taken during the suicide attempt, which is in fact not often available in practice. Therefore, we used “mental disorders” as a variable for adjustment to minimize the potential confounding effect. Further studies recruiting more cases, longer follow-up, and population from other nations are needed to confirm our findings, and basic research on the more detailed mechanism relating to the possible association between COP and adrenal insufficiency is also warranted.

## Conclusion

This nationwide population-based cohort study showed that the risk for developing adrenal insufficiency elevated after COP, especially in the female and patients under 50 years old. The COP patients who had ARF had an even higher risk for developing adrenal insufficiency than those without ARF. The increased risk may be attributable to COP-related hypoxic injury, inflammatory, and immunological reaction on the brain and adrenal gland; however, the underlying mechanism needs further study. It is not unusual that patients with adrenal insufficiency are not diagnosed accurately or not recognized until very sick, such as adrenal crisis. Therefore, close follow-up the adrenal function is recommended in the COP patients, especially in females under 50 years of age during the first year after COP.

## Supplementary Information

Supplementary Information.

## Data Availability

The datasets analyzed during the current study are not publicly available due to the legal restrictions imposed by the government of Taiwan in relation to the “Personal Information Protection Act.” Please contact Chung-Han Ho (2^nd^ author) if someone wants to request the data from this study.

## Abbreviations

COP: Carbon monoxide poisoning
AHR: Adjusted hazard ratio
CI: Confidence interval
CO: Carbon monoxide
NHIRD: National Health Insurance Research Database
NPD: Nationwide Poison Database
LHID2000: Longitudinal Health Insurance Database 2000
ICD-9-CM: International Classification of Diseases, Ninth Revision, Clinical Modification
ATC: Anatomical Therapeutic Chemical
ACTH: Adrenocorticotropic hormone
NTD: New Taiwan dollars
CRH: Corticotropin-releasing hormone
IRR: Incidence rate ratio
