# Supplementary material for: Association between carbon monoxide poisoning and adrenal insufficiency: a nationwide cohort study
Source: Sci Rep. 2022 Sep 28;12:16219. doi: 10.1038/s41598-022-20584-y (PMC9519538; doi:10.1038/s41598-022-20584-y)
Supplement: Supplementary file 1 — Supplementary Information. [file 41598_2022_20584_MOESM1_ESM.docx]

**Supplementary Table 1.** Comparison for inpatient and outpatient visit within 1 year of follow-up between COP and reference cohorts

| Variable | COP (n = 21842) | Reference (n = 43684) | *P*-value |
| --- | --- | --- | --- |
| Within 1 year of follow-up |  |  |  |
| Inpatient visit, yes |  |  |  |
| N (%) | 5251 (24.0) | 3205 (7.3) | <0.001 |
| Inpatient visit, times |  |  |  |
| Mean (standard deviation) | 1.8 (1.7) | 1.4 (1.2) | <0.001 |
| Outpatient visit, yes |  |  |  |
| N (%) | 20169 (92.3) | 40801 (93.4) | <0.001 |
| Outpatient visit, times |  |  |  |
| Mean (standard deviation) | 17.0 (16.8) | 13.2 (13.1) | <0.001 |

COP, carbon monoxide poisoning.

**Supplementary Table 2.** Comparison of adrenal insufficiency between COP patients with ARF and COP patients without ARF

| COP cohort | All-cause mortality  N (%) = 2826 (12.9) | Adrenal insufficiency  N (%) = 53 (0.2) | Crude HR  (95% CI) | AHR  (95% CI)† |
| --- | --- | --- | --- | --- |
| ARF |  |  |  |  |
| Yes | 493 (30.8) | 9 (0.6) | 2.6 (1.3–5.3) | 2.1 (1.0–4.5)^a^ |
| No | 2333 (11.5) | 44 (0.2) | 1 (reference) | 1 (reference) |

COP, carbon monoxide poisoning; ARF, acute respiratory failure; HR, hazard ratio; AHR, adjusted hazard ratio; CI, confidence interval. ^a^*p*=0.048. †Adjusted for sex, underlying comorbidities including liver disease, thyroid disease, mental disorder, and monthly income.

**Supplementary Table 3.** Comparison of adrenal insufficiency between male and female COP patients with ARF

| COP cohort | ARF | | |
| --- | --- | --- | --- |
|  | Male | Female | *P*-value |
| Number of COP patient | 950 | 651 |  |
| Adrenal insufficiency, N (%) | 5 (0.53) | 4 (0.61) | 0.817 |

COP, carbon monoxide poisoning; ARF, acute respiratory failure.

**Supplementary Table 4**. Comparison of the risk for adrenal insufficiency during whole the follow-up period among the COP cohort with different treatment area using competing risk survival analyses

| Treatment area | N (%) | All-cause mortality | Adrenal insufficiency | Crude HR (95% CI) | AHR (95% CI)† |
| --- | --- | --- | --- | --- | --- |
| Overall | 21842 (100.0) | 2826 (12.9) | 53 (0.2) |  |  |
| Outpatient | 14664 (67.1) | 1581 (10.9) | 32 (0.2) | Reference | Reference |
| Hospitalization | 3830 (17.6) | 484 (12.6) | 10 (0.3) | 0.9 (0.4−1.8) | 0.9 (0.5−1.9) |
| ICU admission | 3348 (15.3) | 747 (22.3) | 11 (0.3) | 1.3 (0.6−2.6) | 1.1 (0.6−2.2) |

COP, carbon monoxide poisoning; HR, hazard ratio; AHR, adjusted hazard ratio; CI, confidence interval; ICU, intensive care unit. The definition of “ICU admission” was that patient had any admission of ICU for COP.
